# A new device-aided cognitive function test, User eXperience-Trail Making Test (UX-TMT), sensitively detects neuropsychological performance in patients with dementia and Parkinson’s disease

**DOI:** 10.1186/s12888-018-1795-7

**Published:** 2018-07-05

**Authors:** Naomi Kokubo, Yuma Yokoi, Yuji Saitoh, Miho Murata, Kazushi Maruo, Yoshitake Takebayashi, Issei Shinmei, Sadanobu Yoshimoto, Masaru Horikoshi

**Affiliations:** 1 Translational Medical Center, National Center of Neurology and Psychiatry, 4-1-1 Ogawa-Higashi, Kodaira, Tokyo, 187-8551 Japan; 2 National Center for Cognitive Behavior Therapy and Research, National Center of Neurology and Psychiatry, Tokyo, Japan; 3 Interdisciplinary Graduate School of Medicine and Engineering, University of Yamanashi, Yamanashi, Japan; 4 Department of Psychiatry, National Center Hospital, National Center of Neurology and Psychiatry, Tokyo, Japan; 5 Department of Neurology, National Center Hospital, National Center of Neurology and Psychiatry, Tokyo, Japan; 6 Faculty of Medicine, University of Tsukuba, Ibaraki, Japan; 7 School of Medicine, Fukushima Medical University, Fukushima, Japan; 8 National Institute of Technology, Tokyo College, Tokyo, Japan

**Keywords:** Application, Cognitive function, Dementia, Parkinson’s disease, Screening

## Abstract

**Background:**

A newer generation neuropsychological tests can take advantage of touch screen and mobile technology. We have developed a new Android application termed “User eXperience-Trail Making Test (UX-TMT)” for neurocognitive assessment and training. This study investigated the utility, including the reliability and the validity, of the UX-TMT as a screening test for cognitive decline in adults.

**Methods:**

A total of 84 individuals aged 27–86 years were divided into three groups; healthy controls ([HC] *n* = 29), people with Parkinson’s disease (PD; *n* = 28), and people with mild cognitive impairment (MCI) and dementia (MCI&D; *n* = 27). We examined the distributions of the scores and the time required, and the effects of age and group on these distributions. We analyzed internal consistency and convergent validity in all samples and applied receiver operator characteristic (ROC) analysis to determine a cutoff score that could differentiate the MCI & D group from the HC group.

**Results:**

97.6% of the participants completed all of the tasks, and the average total test time required for UX-TMT was 428.8 (± 109.1) s in the HC, 542.0 (± 168.7) s in the PD, and 777.5 (± 256.1) s in the MCI&D groups, respectively. The MCI&D group showed significantly lower UX-TMT scores and longer total time in completing the task than the HC group. In an ROC analysis, a score of 21 showed high sensitivity (.83) and specificity (.92), and the UX-TMT score plus age improved sensitivity to .96. Additionally, the UX-TMT scores showed significant correlation with the Mini-Mental State Examination (Japanese version) scores (*r* = .77, *p* = .001), and Cronbach’s alpha (.71–.83) indicated acceptable internal consistency.

**Conclusion:**

The UX-TMT demonstrated high reliability and validity to detect cognitive decline in Japanese adults, highlighting its utility as a screening tool for epidemiological and clinical research.

## Background

The number of individuals over 60 years of age worldwide is expected to increase from 901 million in 2015 to more than 2 billion by 2050 [1]. As populations age, age-related neurodegenerative diseases, such as Alzheimer’s disease (AD) and Parkinson’s disease (PD), are expected to increase as well [2].

AD is the most common neurodegenerative disease and the most prevalent form of dementia. The age-standardized prevalence of dementia for those aged ≥60 years varies only slightly across the globe (5–7%), and the prevalence is reported to double in about every 5 years. It has been estimated that 35.6 million people worldwide lived with dementia in 2010, with this number is expected to almost double every 20 years, to reach 115.4 million by 2050 [3]. Neuropathologically, AD is characterized by aggregation and deposition of beta amyloid (Aβ) peptide in the form of neuritic plaques, and hypophosphorylated tau protein in the form of neurofibrillary tangles. Clinically, it is characterized with a progressive loss of cognitive functions, primarily memory [4].

Mild cognitive impairment (MCI) is generally considered as an intermediate state between normal cognitive aging and dementia. Severe cognitive deterioration is often preceded by a preclinical stage with only subtle cognitive deficits that transit to full-blown presentation over time [5, 6]. The definition of MCI has evolved over the past decades. Originally, MCI was considered to show clear memory impairment and taken as prodromal AD. The definition of MCI does not focus sorely on memory impairment but also on impairment in other cognitive domains [7], to be currently classified into 4 subtypes: single-domain amnestic MCI, single-domain non-amnestic MCI, multiple-domain amnestic MCI, and multi-domain non-amnestic MCI [6]. According to the major population-based studies, the average prevalence of MCI is 18.9% based on the expanded Mayo Clinic criteria [7]. In a recent systematic review, the annual conversion rate of MCI to AD is reported to range from 5 to 17% depending on the study sample [8].

PD is the second common neurodegenerative disease after Alzheimer’s disease. In an epidemiological study, the projected number of individuals with PD over age 50 years in 15 of most populous nations was between 4.1 and 4.6 million in 2005, which is expected to double to 8.7–9.3 million by 2030 [9]. PD has been defined primarily as a movement disorder, with typical symptoms being resting tremor, rigidity, bradykinesia, and postural instability; it is pathologically characterized by degeneration of nigrostriatal dopaminergic neurons and the presence of Lewy bodies (misfolded α-synuclein). Recently, cognitive impairment has been increasingly recognized as one of the important non-motor symptoms in PD [10]. In the International Parkinson and Movement Disorders Society (MDS) task force’s first review, the mean prevalence of PD with MCI (PD-MCI) in non-demented patients is 27% (range: 19–38%), and it is associated with subsequent development of PD with dementia (PDD) [11, 12].

Furthermore, PD patients have a much higher cumulative risk of dementia than the age-referenced general population. The incidence of dementia in PDD is increased by 4 to 6 times, and the point prevalence of dementia in PD is close to 30% [13]. Several long-term longitudinal studies have indicated that the majority of patients with PD will develop dementia if they survive for more than 10 years after the diagnosis [10]. In a recent cohort study, the conversion rate from PD-MCI to dementia was 59% at 1 year, although a total of 28% of patients with baseline PD-MCI had reverted to normal cognition at 5-year follow-up [14]. The most common characteristic of PD-MCI is a more rapid decline in cases with non-amnestic and single-domain impairment. In particular, impairments in executive, attentional, and visuospatial domains are notable [10, 11]; behavioral symptoms, such as affective changes, hallucinations, and apathy are frequent as well [13].

Thus, in both AD and PD, cognitive impairments represent one of the major clinical challenges and strongly affect patients’ functioning, Quality of Life (QoL), health-related costs and caregiver’s burden. Although much effort has been made in the development of medication for dementia, the results have been somewhat futile thus far. Accordingly, the interest has been shifted to preventative measures in recent years. If an intervention could delay both disease onset and progression by a modest 1 year, there would be nearly 9.2 million fewer cases by 2050 [15]. In cases of AD, several risk factors (e.g., hypertension, obesity, diabetes, smoking, physical inactivity, and depression) are modifiable [16, 17]. Further, a systematic review and meta-analysis showed that cognitive training leads to measurable improvements in cognitive performance in PD patients, particularly in working memory, executive functioning, and processing speed, which are typically impaired in this disorder [18, 19].

Recently, a series of tablet- and smartphone-based applications for the assessment of cognitive function are appearing. In most previous studies, the utility of these applications has been tested against conventional methods, with a use of intra- and inter-person consistency [20–23]. It has been unclear thought whether these assessments can be completely self-administered by any person [20–24]. Some studies have investigated a novel model or isolated components of cognitive processes [25, 26]. Better assessment strategies would be beneficial both to patients and physicians, potentially minimizing sample size needed in randomized controlled trials (RCTs) by capturing subtler changes during asymptomatic disease stages [27].

We have previously developed a new Android application, the “User eXperience-Trail-Making Test (UX-TMT),” as an assessment and training tool for cognitive function [28]. In this study, we further incorporated 3 other tasks and 3 questions to the UX-TMT for dementia screening. User experience (UX) is one of the key concepts of user-centered approach and is critical in user interface (UI) development, as it comprehensively encompasses user needs, values, abilities, and limitations [29]. The main features of the UX-TMT were that (1) it employs a user-friendly device and design, (2) users are able to work on the tasks in a comfortable way, and (3) it allows clinicians and researchers to objectively and quantitatively evaluate cognitive functions.

We hereby report on discriminating performance of the UX-TMT as a screening test for cognitive decline in Japanese adults.

## Methods

### Participants

People with MCI and dementia (MCI&D), people with PD, and healthy controls (HC) were included in the present study. All participants were recruited through flyers from the National Center of Neurology and Psychiatry (NCNP) in Japan. The inclusion criteria for the MCI&D and PD groups were: a diagnosis of MCI&D or PD by a skilled psychiatrist or neurologist, and the ability to voluntarily understand the aim, risk, and benefit of this study. The exclusion criteria for both groups were: 1) comorbidity with other neurological and/ or psychiatric diseases, 2) noncompliance with the testing, 3) visual decline, hypoacusis, and motor deficits that disturb task performance. The inclusion criteria for the HC group were: over 20 years of age, being healthy, and voluntary participation in this study. The exclusion criteria for the HC group were same with the MCI&D and PD groups. We confirmed their visual, auditory, and motor ability with instructions before implementing the UX-TMT. Neuropsychological tests were individually administered by an experienced clinical psychologist (N.K.).

### Measures

#### The user eXperience-trail making test (the UX-TMT)

The User eXperience-Trail Making Test (UX-TMT) was developed for neurocognitive assessment and training in 2015 [28]. It was inspired by the Advanced Trail Making Test (ATMT), Stroop test, and Reverse Stroop test [25, 30]. These tasks aim to evaluate the function of visuospatial working memory, executive function, and interference inhibition quantitatively. The novel feature of the UX-TMT is a modified trail making test, which includes targets flowing on the tablet screen. These conditions aim to evaluate function of visuospatial cognition and coordinated movement in a quantitative manner. The trail making test (TMT) is among the most commonly-used neuropsychological tests because of its sensitivity to detect structural and functional brain damage. The UX-TMT consists of 3 main components: 1) neurocognitive assessment, 2) cognitive training, and 3) lifelogging. In this paper, we focus on neurocognitive assessment unit, which originally included 6 variations of the TMT. We subsequently added further 4 tasks for comprehensive assessment of cognitive function in adults. The following items make up the revised UX-TMT; these are presented as text on tablet screen and/or by audio.

##### Item 1, demographic information

The participants enter their age, sex, and handedness. When the participant touches input field, a liquid crystal keyboard appears on the screen. After the participant hits enter key, screen transition occurs to fill information.

##### Item 2, mood

The participants enter their current mood in a Likert scale (1: extremely bad, 2: bad, 3: slightly bad, 4: neither bad nor good, 5: slightly good, 6: good, 7: extremely good).

##### Item 3, physical condition

The participants enter their current physical conditions in a Likert scale (1: extremely good, 2: good, 3: neither good nor bad, 4: bad, 5: extremely bad).

##### Item 4, sleepiness

The participants enter their current state of sleepiness in a Likert scale (1: feel smart and wide awake, 2: awake, 3: neither awake nor sleepy, 4: slightly sleepy, 5: extremely sleepy).

##### Item 5, Modified Trail making test

The trail making test (TMT) is a well-known for investigating cognitive performance that depends on executive function (EF), specifically, sustained attention and set-shifting. Recently, advanced trail making test (ATMT) has been developed for quantitative evaluation of visuo-spatial working memory [25]. In the ATMT, we use touch sensor screen instead of paper. The Numbers and Japanese characters “Hiragana” are arranged on the screen as touchable buttons. The ATMT consists of two conditions for both TMT-A and TMT-B. The first is “buttons that are fixed in location (Fixed)” and the second is “buttons that are random in location (Randomized)”. In this study, we add the third condition “buttons that are floating in location (Floating)” for both tests.

**Fig. 1:**
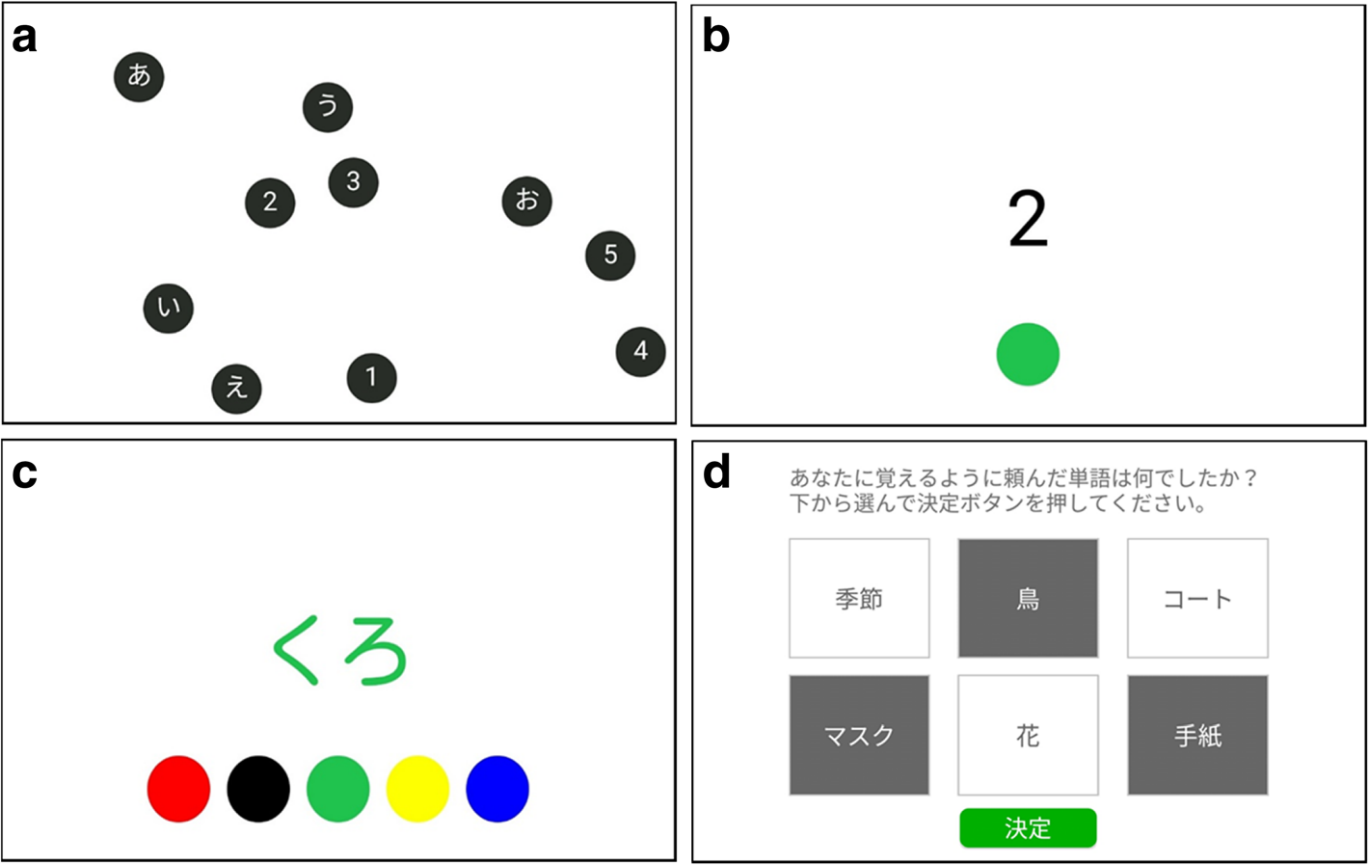
Schematic illustration of cognitive tasks on UX-TMT on 10.1-in. android tablet. **a** TMT-B, “Fixed”, “Randomized”, and “Floating” condition on item 5. 5 digits and 5 Japanese hiragana characters on the screen, **b** Single digit and a single button on item 6, **c** The name of color and 5 colored button on item 7, and **d** Instructing sentence on the top, a set of 6-choice words in the middle, and enter button on the bottom on item 8

In this study, 10 numbers (1, 2, 3, 4, 5, 6, 7, 8, 9, and 10) or 5 numbers and 5 Japanese characters Hiragana ([a], [i], [u], [e], and [o]) are presented at random positions on the tablet screen as buttons (Fig. 1a). In the TMT-A, the participants are instructed to touch the numbers on the screen from 1 to 10 in a sequential order as quickly and accurately as possible. In TMT-B, the participants are instructed to touch alternately between the numbers and hiragana characters in a sequential order (1 - [a] then 2 - [i] through 5 - and [o]), as quickly and accurately as possible. In “Fixed” condition, the location of buttons is the same throughout a single trial, where the participants are able to memorize the position of buttons. When they take advantage of their visuospatial working memory, reaction time can be shortened. In “Randomized” condition, the location of all buttons is changed at random when the correct response has once been made; the participants should search for the following target each time. In “Floating” condition, all buttons are floating during the single trial. Here, the participants are required to make use of their eyes and dominant finger, thus aiming to investigate coordinated movement, sustained attention, and set-shifting. The score in this task ranges from 0 to 12.

##### Item 6, 1-back task

This follows the immediate recall session of the verbal memory task. Single numbers (randomly chosen from 0 to 9) and a single button are presented via text on the screen one at a time (Fig. 1b). Then, each digit is presented for 1.5 s and changed at a fixed inter-stimulus interval of 0.5 s. The participants are instructed to touch the button when the digit is the same as the previous digit, as quickly and accurately as possible. The target is available in a 20% frequency. The score in this task ranges from 0 to 2.

##### Item 7, Stroop and reverse-Stroop (R-Stroop) task

This follows a 1-back task. In this task, 1 word that is the name of a color and 5 colored buttons are displayed on the screen simultaneously (Fig. 1c). The participants are instructed to touch the colored button that matches the font color of the central word. Conversely, in the R-Stroop task, the participants are instructed to touch the colored button that matches the meaning of the central word. There are 2 conditions; one is color-meaning congruence, the “neutral” condition, and the other is color-meaning incongruence, the “interference” condition. In both the Stroop and R-Stroop tasks, the interference condition occurred in a 33.3–41.7% frequency, and each word is presented for 1.5 s and is changed with a fixed inter-stimulus interval of 0.5 s. The participants are instructed to perform the task as quickly and accurately as possible. The Stroop and R-Stroop tasks are a well-known for investigating selective attention to specific information and inhibition of prepotent responses during decision-making tasks involving stimuli and responses [30]. The score in this task ranges from 0 to 6.

##### Item 8, verbal memory task

This task is composed of immediate recall task and delayed recognition task. The former task follows a trail making test. Before the task starts, the participants are instructed to remember 3 words and recall them immediately after they disappear. After the participants enter start button, 3 words are presented on the screen, 1 at a time. If the participants succeed in the immediate recall task, they are asked to recall them later. After the participants finish the R-Stroop task, they are asked to select remembered words from a set of 6 choices on the tablet screen (Fig. 1d). The score in this task ranges from 0 to 6.

In this study, the total UX-TMT score was calculated by summing up the individual score in items 5, 6, 7, and 8, leaving a possible score range from 0 to 26; a higher score indicates a better cognitive performance.

#### The mini-mental state examination-Japanese (the MMSE-J)

The Mini-Mental State Examination is widely used for screening cognitive impairment and decline in about 10 min [31]. The MMSE Japanese version (MMSE-J) has been validated with a high sensitivity and specificity for dementia [32]. In order to avoid practice effects, it was done at least 2 weeks apart from the last examination. The scores in the MMSE-J and those in the UX-TMT were interpreted as an indicator for discriminating validity.

#### The Japanese version of the Montreal cognitive assessment (the MoCA-J)

The Montreal Cognitive Assessment (MoCA) is a brief, well-validated cognitive screening tool for detecting MCI with high sensitivity and specificity [33]. The Japanese version of the MoCA-J is a commonly used screening instrument that was designed to address some of the limitations in the MMSE [34]. In this study, 3 tasks from the MoCA-J were adopted for evaluating executive function and visuospatial abilities (Trail Making B task, a three-dimensional cube copying, and a crock-drawing task). The performance between Trail Making B task in the MoCA-J and the UX-TMT was compared.

#### The 12 item short-form healthy survey (SF-12)

The 12-item short-form health survey (SF-12) is a self-administered questionnaire, commonly utilized to evaluate health-related QOL. They evaluate 2 summary components (physical and mental health) and 8 profiles (physical functioning, role physical, bodily pain, general health, vitality, social functioning, role emotional, and mental health) [35]. In this paper, we focused on the score of vitality in the SF-12 as a comparator to the items 3 and 4 in the UX-TMT.

#### The Japanese version of the 20-item positive and negative affect schedule (PANAS20-J)

The Positive and Negative Affect Schedule (PANAS) is a self-report form and consists of 10 items each for positive affect (strong, inspired, active, enthusiastic, interested, excited, proud, alert, determined, and attentive) and negative affect (afraid, scared, upset, ashamed, guilty, nervous, distressed, irritable, jitter, and hostile) [36]. The Japanese version (PANAS20-J) has been validated and was found to be reliable [37]. We investigated differences across the groups as a reference to the items 2 in the UX-TMT.

A trained clinical psychologist administered the MMSE-J and MoCA-J. The SF-12 and PANAS20-J were self-rated. The UX-TMT is a semi self-administered test because its tutorials are displayed on the screen with the text as an instruction by the examiner. These instruments were administrated in a systematically counterbalanced manner to cancel order effect.

### Statistical analysis

We used χ^2^ tests and two sample t-tests (Welch) to compare demographic and neuropsychological data among the groups. To confirm the effect of age and group on cognitive and other neuropsychological indices, we performed ANCOVA with age as a covariate. Furthermore, we performed receiver operating characteristic (ROC) curve analysis on the MMSE-J scores and UX-TMT scores between the MCI&D and HC groups, to investigate discriminating ability and to determine the cutoff score that is most appropriate. We also performed Spearman’s correlation analysis for the UX-TMT and performance in other neuropsychological tests in order to assess external validity. Finally, Cronbach’s alpha was obtained as an index for internal consistency of the scale.

### Ethical considerations

Participants received verbal and written descriptions of the study in detail and provided their written informed consent. The study protocol was approved by the NCNP Institutional Review Board.

## Results

In total, 84 participants were assigned either to the MCI&D (*n* = 27), PD (*n* = 28), or HC (*n* = 29) groups, respectively. Three participants in the MCI&D group were excluded from analysis, because the diagnosis was altered in one patient and two could not complete the UX-TMT. Demographic and neuropsychological characteristics of the participants are summarized in Table 1. There were no significant differences in sex and handedness among the 3 groups. The MCI&D group had significantly fewer years of education than the HC group. The HC group was significantly younger than the MCI&D and PD groups. Within the HC group, the lowest score in the MMSE-J was 24.

**Table 1:** Demographic characteristics of participants

	HC	MCI&D	PD	
	Mean (SD)	Mean (SD)	Mean (SD)	Welch’s t-test *p*-value
Age	55.8 (13.7)	79.4 (7.6)	68.6 (6.7)	< 0.001
Sex (Male:Female)	11:18	13:11	13:15	0.516
Handedness (Left:Right)	1:28	1:23	2:16	0.532
Education (y)	15.5 (3.8)	13.8 (2.3)	14.4 (2.5)	0.209
Mood (item 2)	4.9 (1.0)	4.7 (1.5)	4.9 (1.0)	0.883
Physical Condition (item 3)	2.3 (0.8)	3.0 (1.1)	2.8 (1.0)	0.026
Sleepiness (item 4)	2.5 (1.3)	2.5 (1.1)	3.4 (1.0)	0.004
PANAS20-J_P	32 (7.7)	29.4 (6.3)	29.29 (6.5)	0.155
PANAS20-J_N	19.5 (6.5)	22.7 (8.0)	24.8 (7.3)	0.005
SF12_Vitality	54.1 (11.1)	51.0 (11.3)	48.2 (11.8)	0.056

MMSE-J: Mini-Mental State Examination Japanese, MoCA-J: Japanese version of the Montreal Cognitive Assessment, EF: executive function, TMT: Trail Making Test, PANAS20-J_P: The Japanese version of 20-item Positive and Negative Affect Schedule positive affect score, PANAS20-J_N: The Japanese version of 20-item Positive and Negative Affect Schedule negative affect score, SF12_Vitality: SF-12 healthy survey, score on the subscale of vitality

### Reliability

Cronbach’s alpha for the UX-TMT fell into a range of 0.71–0.83 in all 3 groups, indicating good internal consistency across the sample.

### Validity

#### Discriminative validity

**Fig. 2:**
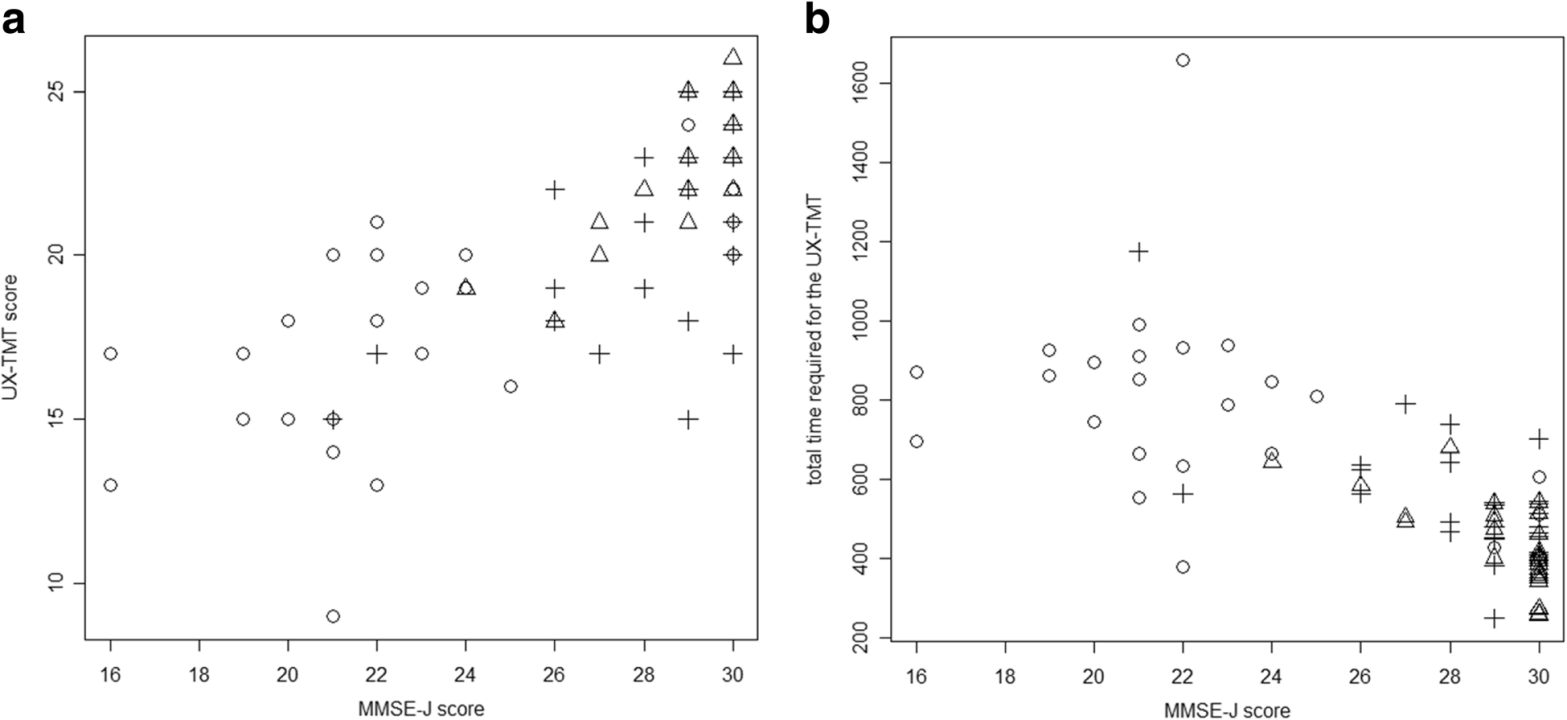
Distribution of UX-TMT score, total test time and MMSE-J score. **a** Distribution of UX-TMT score against MMSE-J score, and **b** distribution of total test time for UX-TMT and MMSE-J score. Triangle: Healthy Control (HC), cross: Parkinson’s disease (PD), circle: Mild Cognitive Impairment & Dementia (MCI&D), (*n* = 81)

Figure 2 shows scatter plot of the UX-TMT scores (A), the total time required (B) and the MMSE-J score distributions among the 3 groups. Because of the significant differences in age between the 3 groups, we performed ANCOVA for the cognitive tests and the results are shown in Table 2. As expected, people in the MCI&D group performed worst in terms of cognitive assessments. Cognitive performances in the PD group fell within those in the MCI&D and HC groups, without statistical differences from the latter group.

**Table 2:** Cognitive outcomes

	HC	MCI&D		PD	
	Mean (SD) [range]	Mean (SD) [range]	ANCOVA age-adjusted p-value	Mean (SD) [range]	ANCOVA age-adjusted p-value
UX-TMT score	23.2 (2.0)	17.6 (3.4)	0.003	21.0 (2.9)	0.260
UX-TMT_TMTB	1.8 (0.4)	1.5 (0.5)	0.217	1.8 (0.4)	0.458
UX-TMT_TRT (sec)	98.0 (38.2)	251.7 (112.2)	0.010	166.4 (106.5)	0.192
UX-TMT_TTT (sec)	428.8 [255.9–678.4]	777.5 [379.3–1658.5]	0.004	542.0 [248.0–1173.3]	0.520
UX-TMT_B-A (sec)	1.0 (1.3)	3.6 (3.2)	0.071	2.1(3.1)	0.365
MMSE-J	29.2 (1.5)	22.5 (3.9)	< 0.001	28.3 (2.3)	0.721
MoCA-J_EF	4.7 (0.5)	3.7 (1.3)	0.008	4.7 (0.6)	0.835
MoCA-J_TMT	1.0 (0.2)	0.5 (0.5)	0.004	0.9 (0.3)	0.881

Mood: The score of current mood on the UX-TMT, Physical Condition: The score of current physical condition on the UX-TMT, Sleepiness: The score of current state of sleepiness on the UX-TMT, UX-TMT score: Total score of the User experience-Trail Making Test, TMTB: The score of Trail Making Test-part B on UX-TMT, TRT: total response time, TTT: total test time, TMT_B-A: the difference of mean reaction time between TMT-part A and -part B on the UX-TMT

**Fig. 3:**
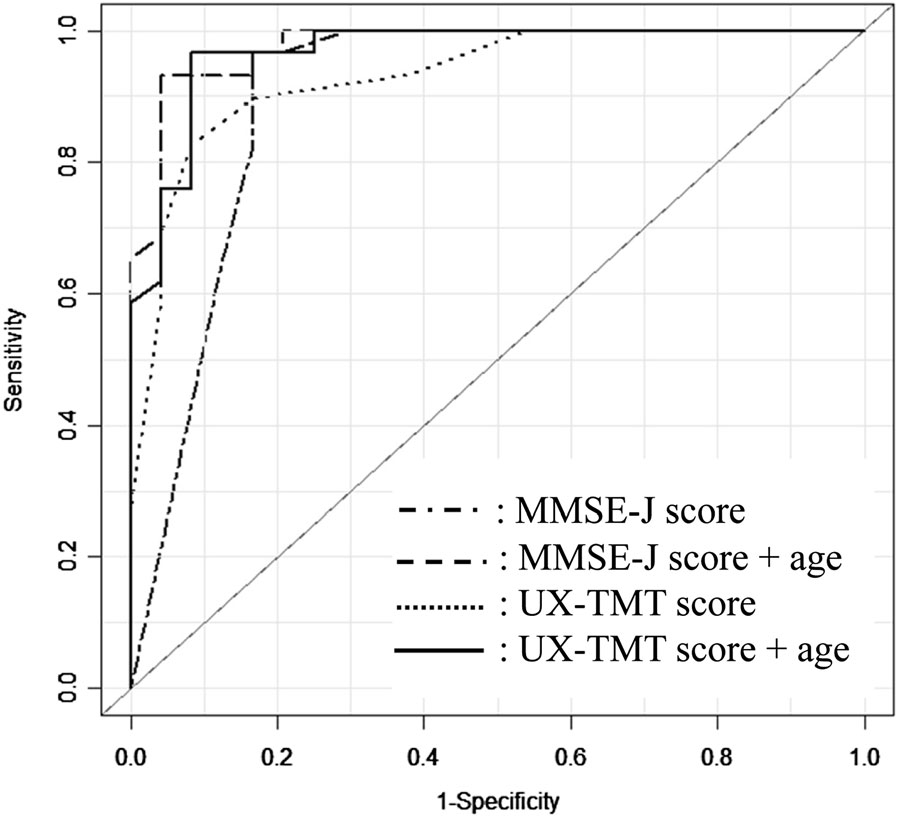
Receiver Operating Characteristic curve with UX-TMT or MMSE-J scores and age for differentiation. To differentiate HC from MCI&D groups (*n* = 53), a score of 21 in UX-TMT showed high sensitivity (.83) and specificity (.92), and UX-TMT score plus age improved sensitivity to .96. Long and short dashed line: MMSE-J score, long dashed line: MMSE-J score plus age, dotted line: UX-TMT score, solid line: UX-TMT score plus age

To verify discriminating ability of the UX-TMT, we performed an ROC analysis on the MMSE-J and UX-TMT scores in the MCI&D and HC groups (*n* = 53). Figure 3 indicates that, at a score of 21, the area under the curve was 0.93, and the sensitivity and specificity were 0.82 and 0.91, respectively. A total of 48 subjects, 4 in MCI&D group, 18 in PD group, and 26 in HC group, respectively, had a score of more than 21 in the UX-TMT. When the patients’ age was added as an explanatory variable on the UX-TMT score, the area under the curve improved to 0.97, and the sensitivity and specificity were 0.97 and 0.92, respectively. In reference, the MMSE-J score of 25 demonstrated the area under the curve of 0.902, with the sensitivity and specificity being 0.97 and 0.83, respectively. When the patients’ age was added as an explanatory variable on the MMSE-J score, the area under the curve, sensitivity and specificity were 0.98, 0.93 and 0.96, respectively.

#### Convergent validity

The results of correlational analysis among the UX-TMT indices, age, and other neuropsychological tests are summarized in Table 3. The UX-TMT scores had a strong correlation with the MMSE-J scores (*r* = 0.77, *p* < .001). Furthermore, the Trail Making Test part B (TMT-B) score in the UX-TMT showed a moderate correlation with the TMT-B score in the MoCA-J (*r* = 0.56, *p* < .001) (Table 3).

**Table 3:** Correlation coefficients among variables

	UX-TMT score	UX-TMT_ (table-based) TMT-B	UXTMT_Total Test Time: TTT	Age
Age	−0.68	−0.33	0.62	–
MMSE-J	0.77	0.49	−0.71	−0.62
MoCA-J_EF	0.51	0.52	−0.46	− 0.34
MoCA-J_TMT	0.64	0.56	−0.57	−0.40

MMSE-J: Mini-Mental State Examination Japanese, MoCA-J EF: Executive function (EF) aspects score on the Japanese version of the Montreal Cognitive Assessment, MoCA-J_TMT: Trail Making Test score on the Japanese version of the Montreal Cognitive Assessment, UX-TMT score: scores on the User experience-Trail Making Test, UX-TMT_(tablet-based) TMT-B: score on mode 2 of item 5 on the UX-TMT (TMT task-B)

## Discussion

Recent studies have suggested that preventative intervention should start at the earliest opportunity. However, these subclinical individuals often pass currently available screening tests, such as the Mini-Mental State Examination (MMSE), and the Montreal Cognitive Assessment (MoCA), underscoring a need to develop new measures with high sensitivity and specificity to accurately detect preclinical status. In recent years, there has been an interest in identifying subjective cognitive decline (SCD) as an indication to start preventative interventions. SCD is a situation where cognitive impairments are identified by the patients, family members, or healthcare personnel, even though the patient’s cognitive test performance is in the “normal” range [8]. In the general population, SCD is associated with an increased risk of future cognitive decline, that is, progression to MCI or dementia including AD [10]. However, there have been few validated and reliable methods for capturing SCD. Therefore, a user-friendly neurocognitive and behavioral assessment tool is crucial that can be applied to high-frequency in-home monitoring [20].

To this end, we revised the UX-TMT for the purpose of screening cognitive impairment in adults in a cross-diagnostic manner. This report provides some evidence on its utility, validity, and reliability. The time required was short enough to be user-friendly; the average total test time was 428 to 778 s across the groups, with modest standard deviations. Some major screening tests for cognitive impairment, e.g., the MMSE-J, the MoCA-J, and Hasegawa’s Dementia Scale-Revised require from 6 to 10 min to complete [38]. One of the main features of the UX-TMT is that we can record detailed time required for each of the procedure.

In this study, this semi self-administrated instrument indicated good acceptability/tolerability in that 97.6% of the participants completed all of the tasks. In the SMART study, 65% of the cognitively intact participants and merely 29% of the MCI participants were able to complete a computer-based assessment for cognitive function at home [39]. In the ADNI study, Maruff et al. compared between in-clinic and unsupervised assessments for completion rates, and examined age and gender effects, effect of diagnoses as well as stability over time. They reported that 98% of a total 100 participants [cognitive normal = 51, mild cognitive impairment (MCI) = 41, Alzheimer’s disease dementia (AD) = 6] completed baseline in-clinic assessment. The completion rate of second unsupervised assessment was down to 78%, with 92% requiring one attempt [40]. These results call for a user-friendly assessment scales that are cross-diagnostic and can be used in unsupervised settings for the elderly people whose tolerability to tests may well be compromised.

The UX-TMT scores and the total time required for the UX-TMT were significantly different between the MCI&D and HC groups. The ROC analysis (*n* = 53) indicated that the UX-TMT showed a high sensitivity (0.83) and specificity (0.92) at a cutoff score of 21. When age was added as an explanatory variable, the predictive performance improved. The MMSE-J demonstrated almost the same sensitivity (0.97) and specificity (0.83) between the HC and MCI&D groups. It is known that the MMSE scores are affected by age not only in HCs, but also in dementia and PD patients [41–44], and we also added age as an explanatory variable in subsequent analyses; the performance was similar to the UX-TMT. Pedraza et al. reported that age- and education-adjusted MMSE score of 23/24 provided excellent classification accuracy for dementia (0.96), with a high sensitivity (0.92) and specificity (0.98), together with the accuracy (0.96) of unadjusted cutoff score of 22/23 in African American older adults [41]. Onoda et al. has reported that the scores of the cognitive assessment for dementia, iPad version: CADi2, had a high sensitivity (0.85) and specificity (0.81) in participants with moderate AD and age-matched healthy controls [27]. Thus, the UX-TMT was found to be at least equivalent to, or better than, the currently available alternatives.

The PD patients tended to report lower mood and functions in conventional self-rating questionnaires, corroborating the previous study [13]. It is well known that the sleepiness is deeply associated with the cognitive function. Some studies suggest that an electronic diaries or single-item measures are useful for evaluating subjective and objective function [45, 46]. The results suggest that the items in the UX-TMT (i.e., mood, physical condition, and sleepiness) are expected to aid in evaluation of subjective cognitive issues in PD patients briefly. In this study, the UX-TMT did not statistically differentiate PD patients from controls; this may have stemmed from a selected (cognitively preserved) population, limited sample size or a possibility that our scoring method is not sensitive enough.

The UX-TMT showed significant correlations with the MMSE-J (*r* = .77) and the trail making test part B of the MoCA-J (*r* = .56). Fellows et al. reported that the digital trail making test (dTMT) showed significant correlation with paper version of trail making test part A (*r*s = .541) and part B (*r*s = .799), respectively, in 68 participants comprised of the elderly controls and patients including PD and MCI [26]. They administrated the test by using a stylus instead of pencil and used 26 encircled numbers and letters in paper version and 20 or 19 encircled numbers and letters in tablet version TMT, respectively. In our study, we used 10 encircled numbers and Japanese characters Hiragana. Further research may be needed to decide the optimal methodology to deliver TMT. Cronbach’s alpha (0.71–0.83) showed acceptable consistency for the UX-TMT.

The applications of cognitive assessments can be combined with other technologies, such as smartphone and video conferencing, and the feasibility of remote assessment has been verified [24]. Recently, the next generation applications take advantage of touch sensors and mobile technology [25, 30]. They have been applied to exploring behavioral indices and quantitative measurements of perturbed cognitive processes [25, 26, 30, 47]. Other possibility includes real-time and remote behavioral monitoring of cognitive and motor function in the elderly [26, 47]. Larger studies using age- and education-matched sample are required to further test the usefulness of the UX-TMT in senior populations. Possible diurnal variation may be addressed with the event- or time-driven methods. Test-retest reliability of the UX-TMT should be addressed in the subsequent studies. Nonetheless, it is possible that this instrument may serve to patient-centered design for e-healthcare in the future.

## Conclusion

In this novel revision of the UX-TMT, 3 tasks were added to enable more comprehensive assessment for cognitive function. We found that the UX-TMT had high feasibility, sensitivity, and specificity for discriminating between people with MCI and dementia from controls, suggesting adequate construct validity. An optimal cutoff score of 21 was identified to be useful in clinical settings. The UX-TMT scores were significantly correlated with those in the MMSE-J, demonstrating its concurrent validity. The UX-TMT had moderate Cronbach’s alpha values, indicating good internal consistency. The UX-TMT is now only available in Japanese but may be of use as a mass-screening and to monitor disease progression of dementing disorders.

## Abbreviations

AD: Alzheimer’s Disease
ANCOVA: Analysis of covariance
ATMT: Advanced Trail Making Test
dTMT: digital Trail Making Test
EF: Executive Function
HC: Healthy Controls
MCI: Mild Cognitive Impairment
MCI&D: Mild Cognitive Impairment & Dementia
MDS: the international parkinson and Movement Disorder Society
MMSE-J: Mini-Mental State Examination Japanese version
MoCA-J: Japanese version of the Montreal Cognitive Assessment
NCNP: National Center of Neurology and Psychiatry
PANAS20-J: The Japanese version of 20-item Positive and Negative Affect Schedule
PD: Parkinson’s Disease
PDD: Parkinson’s Disease with Dementia
QoL: Quality of Life
RCTs: Randomized Controlled Trials
ROC: Reciever Operating Characteristic
R-Stroop task: Reverse-Stroop task
SCD: Subjective Cognitive Decline
SF-12: The 12 item short-form Healthy Survey
TMT: Trail Making Test
TRT: Total Reaction Time
TTT: Total Test Time
UI: User Interface
UX: User eXperience
UX-TMT: User experience?Trail-Making Test
WM: Working Memory
